# Daidzein Changes Production Performance, Meat Quality, and Transcriptome of Muscle in Heat-Stressed Jinjiang Cattle

**DOI:** 10.3390/ani15182650

**Published:** 2025-09-10

**Authors:** Huan Liang, Kun Fu, Lin Li, Xiaozhen Song, Long Wang, Lanjiao Xu, Mingren Qu

**Affiliations:** Key Laboratory of Animal Nutrition in Jiangxi Province, Jiangxi Agricultural University, Nanchang 330045, China; lianghuan22@163.com (H.L.); 15279852465@163.com (K.F.); li_lin1011@hotmail.com (L.L.); songxz1234@163.com (X.S.); wanglong960627@163.com (L.W.)

**Keywords:** Jinjiang cattle, daidzein, heat stress, growth performance, meat quality, RNA-Seq

## Abstract

This research was carried out to assess the impact of daidzein supplementation on production performance, serum biochemical indexes, meat quality, and the transcriptome of the longissimus dorsi (LM) muscle in heat-stressed Jinjiang cattle. Twenty 20-month-old Jinjiang cattle (initial mean ± SE: 438 ± 34.6 kg of body weight) were randomly divided into two treatment groups (*n* = 10 per treatment): control treatment and daidzein treatment (1000 mg/kg concentrate). After a 100-day feeding trial (consisting of a 10-day adaptation period and a 90-day daidzein feeding period), blood and LM muscle samples were collected on day 100. Daidzein significantly increased the average daily dry matter intake (ADMI), the concentration of free fatty acid (FFA) and glutamic-pyruvic transaminase (ALT) in serum, and the marbling score of the LM muscle. Additionally, daidzein significantly decreased the concentration of total cholesterol (TC) and leptin in serum, along with the shear force and L* value of LM in heat-stressed Jinjiang cattle. The transcriptome analysis demonstrated that 238 differentially expressed genes (DEGs) were identified through differential expression analysis, among which 168 genes were downregulated and 70 genes were upregulated. The results of KEGG pathways showed that these DEGs were significantly enriched in pathways related to beef tenderness, including the FoxO signaling pathway, Notch signaling pathway, and regulation of the actin cytoskeleton. Daidzein significantly affected the candidate genes (*FOSL1*, *DGKH*, *Gadd45G*, *GAL*, *SEMA3*, *TOB*, *FABP8*, *TRIB2*, *Nech1,* and *GSTA3)* involved in adipocyte differentiation, as well as genes (*CSTB* and *ACTN*) related to connective tissue structure in heat-stressed Jinjiang cattle. Daidzein plays a positive role in relieving heat stress and improving beef quality in heat-stressed Jinjiang cattle.

## Simple Summary

The impact of heat stress on beef quality has emerged as a critical challenge for the sustainable and high-quality development of the beef cattle industry. Daidzein has been recognized for its potential to alleviate heat stress and improve meat quality in livestock. However, its effects on meat quality in heat-stressed beef cattle remain largely understudied. This study investigated the effects of daidzein supplementation on production performance, serum biochemical parameters, meat quality, and the transcriptomic profile of the longissimus dorsi muscle (LM) in heat-stressed Jinjiang cattle. The results indicate that daidzein significantly decreased serum concentrations of TC and leptin, as well as shear force and L* values in the LM of heat-stressed beef cattle. Transcriptome analysis revealed that daidzein altered the expression levels of several genes and enriched several signaling pathways in LM of heat-stressed Jinjiang cattle. These findings suggest that daidzein plays a positive role in relieving heat stress and improving beef quality in heat-stressed Jinjiang cattle.

## 1. Introduction

Due to global warming, heat stress has become increasingly prevalent in livestock production [1]. Heat stress adversely affects animal nutritional metabolism, resulting in reduced body weight, impaired intake and feed efficiency, as well as diminished carcass quality, ultimately leading to substantial economic losses [2]. Beef cattle are particularly susceptible to heat stress due to their rapid metabolic rate, rumen fermentation characteristics, impaired sweating capacity, and skin insulation properties [3,4]. Several reports have demonstrated that heat stress can significantly reduce beef quality. Surinder et al. [5] demonstrated that beef cattle subjected to summer heat stress exhibited significantly higher shear force values in the muscles, leading to reduced tenderness and compromised meat quality. Heyok et al. [6] reported that Korean cattle steers exposed to prolonged high-temperature and high-humidity stress showed a marked decline in post-slaughter muscle tenderness, ultimately resulting in substantial deterioration of beef quality attributes. The impact of heat stress on beef quality has emerged as a critical challenge for the high-quality development of the beef cattle industry.

Natural plant extracts contain a rich variety of active ingredients such as isoflavones and polysaccharides [7]. These ingredients have the characteristics of antioxidation and anti-inflammation. Hence, reducing heat stress and improving beef quality by feeding with plant extract additives has been a research hotspot in recent years [8,9]. Soybean isoflavones are the secondary metabolites of polyphenol compounds in soybean products [10]. Daidzein (7,4′-trihydroxyisoflavone) is one of the major soybean isoflavones [11]. Liu et al. [12] reported that supplementing daidzein in the diet significantly enhanced the growth performance, antioxidant capacity, and immune capacity of bull calves. Wang et al. [13] demonstrated that dietary supplementation with isoflavones significantly upregulated both the expression levels and protein abundance of heat shock proteins (HSPs) in the serum of heat-stressed mice. Wang et al. [14] elucidated that isoflavones attenuated heat stress hazards by upregulating HSP70 expression, thereby improving heat stress resilience in caprine species. These results indicate that daidzein can be used to alleviate heat stress in livestock.

Because the chemical structure of daidzein is similar to estrogens, some researchers have reported that daidzein could affect the meat tenderness of livestock. Zhao et al. [15] demonstrated that supplementing daidzein in diet significantly improved beef tenderness by increasing the intramuscular fat content and marbling score in Xiangzhong black cattle. Rehfeldt et al. [16] reported that adding daidzein to the diet significantly affected the meat tenderness of piglets by transforming the muscle fiber types. In our previous study, adding 1000 mg/kg daidzein to the diet significantly increased the fat thickness (1.8 cm vs. 1.4 cm, *p* < 0.05) and significantly decreased the shear force (3.0 kg f vs. 3.5 kg f, *p* < 0.05) of beef in Xianan steers [17]. However, little attention has been paid to the effect of daidzein on meat quality in heat-stressed beef cattle.

This research was carried out to assess the impact of daidzein supplementation on production performance, serum biochemical indexes, meat quality, and the transcriptome of the longissimus dorsi (LM) muscle in heat-stressed Jinjiang cattle, so as to provide a theoretical basis for the application of daidzein in heat-stressed beef cattle production.

## 2. Materials and Methods

### 2.1. Animal Care

These experiments were conducted following Chinese guidelines for animal welfare. All the experimental procedures applied in this study were reviewed and approved by the Committee for the Care and Use of Experimental Animals at Jiangxi Agricultural University (JXAULL−20190015).

### 2.2. Animals, Diets, and Experimental Design

Daidzein (purity > 98%) was acquired from Ciyuan Biotechnology Limited Company (Baoji, Shanxi, China). The cattle were provided by Shenglong Cattle Industry Limited Company (Pingxiang, China). Twenty 20-month-old Jinjiang cattle (initial mean ± SE: 438 ± 34.6 kg of body weight) were randomly divided into two treatment groups (*n* = 10 per treatment): control treatment and daidzein treatment (1000 mg/kg concentrate). The dosage of daidzein supplemented in the diet was based on our previous study, in which adding 1000 mg/kg daidzein to the diet significantly increased the fat thickness and significantly decreased the shear force of beef in Xianan steers [17]. The composition and nutritional content of the basal diet are presented in Table 1. All cattle were housed individually. Diets were fed twice daily at 07:00 and 14:00. Clean, fresh water was provided as needed. The 100-day feeding trial, conducted from July 20 to September 27, included a 10-day adaptation period followed by a 90-day experimental period under controlled environmental conditions in the cattle housing facility, with an averaging temperature of 30.68 °C, relative humidity of 68.05%, and a temperature–humidity index (THI) of 81.82.

### 2.3. Growth Performance and Serum Biochemical Parameters

The individual body weights of all cattle were recorded prior to morning feeding at both the start and end of the trial. Average daily gain (ADG) was calculated as ADG (kg/d) = (final body weight − initial body weight)/trial duration (days). Average daily dry matter intake (ADMI) was determined by ADMI (kg/d) = (feed offered − feed residues)/trial duration (days). Feed residues were collected and weighed daily before morning feeding. The feed conversion ratio (FCR) was derived as FCR = ADMI/ADG.

On the final day of the experiment, blood samples were collected from the caudal vein prior to morning feeding. After standing for 30 min, the blood samples were centrifuged at 3500 revolutions per minute for 15 min. Blood biochemical parameters, including glucose, free fatty acid (FFA), triglyceride (TG), total cholesterol (TC), total protein (TP), albumin, urea, glutamic-oxalacetic transaminase (AST), glutamic-pyruvic transaminase (ALT), low-density lipoprotein cholesterol (LDL-C), high-density lipoprotein cholesterol (HDL-C), total antioxidant capacity (T-AOC), malondiadehyde (MDA), total superoxide dismutase (T-SOD), glutathione peroxidase (GSH-PX), growth hormone (GH), triiodothyronine (T3), tetraiodothyronine (T4), cortisol (COR), leptin, insulin, adiponectin, IgA, IgM, and IgG, were quantified using a Hitachi 3100 automatic biochemical analyzer (China).

### 2.4. Meat Quality

On the final experimental day, four bulls with medium body weight were selected from the control and daidzein groups, respectively. Those eight bulls were slaughtered following commercial slaughter protocols. The bulls were individually stunned in a 260 × 81 × 160 cm (length×width×height) steel-walled pen (stun box). Then, the stunned bulls were slaughtered using upright restraint. All carcasses were immediately chilled at 0 °C for a 7-day aging period. The meat quality was measured from the longissimus dorsi muscle excised from the right-side 12th−13th rib interface and carefully trimmed to exclude connective tissue and subcutaneous fat. The marbling score was evaluated using the Japanese Marbling Standard (JMBS) [18]. The marbling standard consists of 12 grading levels (8–12 = Abundant, 5–7 = Moderate, 3–4 = Average, 2 = Slight, 1 = Trace). The pH value of the longissimus dorsi muscle was determined by a Delta 320 pH Meter (Mettler Toledo, Switzerland), with the probe inserted into the muscle core. Meat color values of L*, a*, and b* were measured by a colorimeter (WSC-S, Shanghai, China) with a D65 illuminant, 10° observer angle, and CIELAB color space.

To determine the cooking loss and shear force values, LM samples were standardized into 3 × 4 × 5 cm steaks, sealed within individual ziplock bags, and cooked in a temperature-controlled water bath (75 °C) until the core temperature reached 70 °C. Then, the cooked LM samples were cooled to room temperature naturally. The cooking losses were calculated as (raw weight–cooked weight)/raw weight ×100%. Six 3 × 1 × 1 cm muscle fiber-aligned subsamples were prepared from each LM and sheared perpendicular to fiber orientation using a C-LM4 device (Harbin, China) with a load cell of 15 kg and a crosshead speed of 200 mm/min. The shear force was expressed as mean peak force (kg·f) from the six replicates. To determine the drip losses, the valve-bag protected LM samples were stored at 4 °C for 48 h, and the fiber orientation was maintained parallel to gravitational force. Drip losses were calculated as (initial weight–refrigerated weight)/initial weight × 100%. The chemical compositions of the basal diet and LM were measured following the procedures of AOAC methods [19].

### 2.5. RNA-Seq Library Preparation and Data Analysis

The total RNA was isolated from eight LM samples by TRIzol reagent (LC Science, Houston, TX, USA) according to the manufacturer’s instructions. The Agilent 2100 Bioanalyzer (Agilent, CA, USA) was used to assess the total RNA quality and concentration. Then, transcriptomic sequencing was performed by Illumina HiSeq™ 2000 (Novogene, Beijing, China).

The samples were removed if raw reads had more than 17% unknown nucleotides. The valid reads of each sample were aligned to the Bos taurus genome assembly (https://ftp.ensembl.org/pub/release-95/fasta/bos_taurus/dna/ accessed on 25 August 2025).

To analyze gene expression, the number of unique-match reads was calculated and normalized to FPKM (fragment per kilo base of exon model per million mapped reads), which was used to indicate the condition of transcriptional expression. The amount of expression was calculated for each read of the eight sequenced samples using Cuffdiff [20].

To determine the functional categories of differentially expressed genes (DEGs), all DEGs were subjected to GO and KEGG pathway analyses. GO enrichment analysis was used to map all DEGs to GO terms in GO database 2. The significance was calculated using a hypergeometric test, as suggested by Yang [8].

To better understand the biological function of DEGs, all DEGs were annotated to KEGG (Kyoto Encyclopedia of Genes and Genomes) pathways.

The original contributions presented in the study are publicly available. This data can be found here: (https://www.ncbi.nlm.nih.gov/Traces/study/?acc=PRJNA676129, access on 13 November 2020).

### 2.6. Real-Time Quantitative PCR (qRT-PCR)

Microbial DNA was extracted from rumen fluid samples using an E.Z.N.A. stool DNA kit (Omega Bio-tek, Norcross, GA, United States) according to the manufacturer’s protocols. The 16S rDNA V3–V4 region of the eukaryotic ribosomal RNA gene was amplified by PCR ( 95 °C for 3 min, followed by 30 cycles at 95 °C for 30 s, 55 °C for 30 s, and 72 °C for 45 s, and a final extension at 72 °C for 10 min (until halted by user)) using primers 338F: ACTCCTACGGGAGGCAGCAG and 806R: GGACTACHVGGGTWTCTAAT. PCRs were performed in a triplicate 20 µL mixture containing 4 µL of 5×FastPfu buffer, 2 µL of 2.5 mM dNTPs, 0.8 µL of forward primer (5 µM), 0.8 µL of reverse primer (5 µM), 0.4 µL of FastPfu polymerase, 0.2 µL of BSA, and 10 ng of template DNA.

PCR products were purified using an AxyPrep DNA Gel Extraction Kit (Axygen Biosciences, Union City, CA, USA) according to the manufacturer’s instructions and quantified using a Quantus™ Fluorometer (Promega, Madison, WI, USA). Purified amplicons were pooled in equimolar ratios and paired-end sequenced on an Illumina NovaSeq PE250 platform (Illumina, San Diego, CA, USA) according to the standard protocols of Majorbio Bio-Pharm Technology Co., Ltd. (Shanghai, China). The raw reads were deposited into the NCBI Sequence Read Archive (SRA) database (Accession Number: PRJNA884686).

### 2.7. Processing of Sequencing Data

To confirm the reproducibility and repeatability of gene expression data obtained by RNA-Seq, eight DEGs in the longissimus dorsi muscle of heat-stressed Jinjiang cattle were selected for qRT-PCR validation. The TRIzol reagent (LC Science, Houston, TX, USA) was used to isolate total RNA. Gene-specific primers were designed according to the gene sequence using Primer 5.0 software (Premier Biosoft, Palo Alto, CA, USA) and synthesized by Takara Bioch (Dalian, China) (Table 2). qRT-PCR was performed with the BioRad PCR machine with SYBR green master mix (TransGen Biotech, Beijing, China) following the manufacturer’s guidelines. The reaction mixtures were incubated in a 96-well plate at 95 °C for 20 s, followed by 40 cycles of 95 °C for 3 s and 60 °C for 30 s. All measurements were analyzed in triplicate. The relative mRNA expression of the eight DEGs was achieved after normalization of glyceraldehyde−3-phosphate dehydrogenase (GAPDH) reference using the 2^−ΔΔCt^ method [21].

### 2.8. Statistical Analysis

The analysis of the experimental data was conducted using SPSS 26.0 (SPSS, Chicago, IL, USA), where independent sample t-tests were employed to assess the significance of production performance, serum biochemical indexes, and meat quality. An independent sample *t*-tests is an important parameter test method in statistics to compare the mean difference of two groups of independent samples. It verifies whether there is a significant mean difference between two non-interfering sample groups by analyzing the distribution characteristics of the two groups of data. The final results are presented as mean values, with differences regarded as showing a tendency when 0.05 < *p* < 0.10 and considered statistically significant at *p* < 0.05.

## 3. Results

### 3.1. Growth Performance

The effects of daidzein on production performance in heat-stressed Jinjiang cattle are presented in Table 3. Compared with the control treatment, the addition of daidzein significantly increased the average daily dry matter intake (*p* < 0.05).

### 3.2. Serum Biochemical Parameters

Table 4 illustrates the impact of daidzein on the serum biochemical indexes in heat-stressed Jinjiang cattle. No significant differences were observed in the serum levels of TG, glucose, TP, albumin, HDL-C, LDL-C, urea, AST, T-SOD, MDA, GSH-PX, T-AOC, IgA, IgM, IgG, T3, T4, COR, GH, insulin, and adiponectin between the control group and the daidzein group. However, the concentrations of TC and leptin in the daidzein group were significantly lower than those in the control group (*p* < 0.05). In contrast, the levels of FFA and ALT were significantly higher in the daidzein group compared to the control group (*p* < 0.05).

### 3.3. Meat Quality

**Figure 1:**
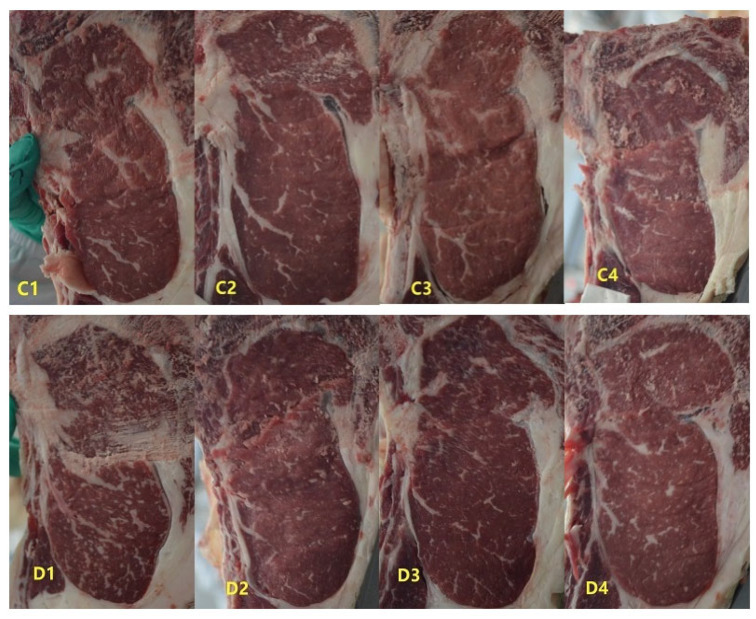
Marbling score between control (**C1**–**C4**) and daidzein (**D1**–**D4**) groups of the longissimus dorsi muscle in heat-stressed Jinjiang cattle.

As shown in Table 5 and Figure 1, the marbling score of LM in the daidzein group was significantly higher than that of the control group (*p* < 0.05). The shear force and L* value of LM in the daidzein group were significantly lower than those in the control group (*p* < 0.05). However, supplementation with daidzein had no significant effect on the pH, drip loss, cooking loss, a*, b*, moisture, ash, crude protein, and intramuscular fat content of LM in heat-stressed Jinjiang cattle (*p* > 0.05, Table 5).

### 3.4. Overall Assessment for Mapping Statistics

**Figure 2:**
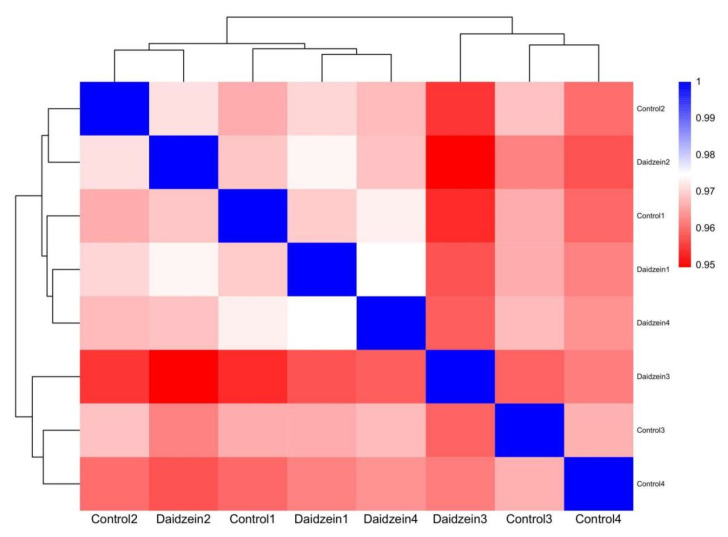
Correlations of eight samples.

The overall mapping statistics are summarized in Table 6. RNA-Seq of eight LM samples generated approximately 42.6 million raw reads. After quality filtering, each muscle sample yielded an average of 4.90 gigabases (Gb) of high-quality sequence data, ranging from 4.64 to 5.46 Gb. Using TopHat2 software for alignment, more than 77.94% of the clean reads per sample were successfully mapped to the reference genome. Of these mapped reads, 74.53–82.77% were uniquely aligned, while 3.41–4.27% were aligned to multiple genomic locations. Correlation analysis based on gene expression profiles revealed that the correlations between the biological replicates were consistently above 0.952 (Figure 2). This high level of reproducibility across the samples indicates that the RNA-Seq data are reliable and suitable for further analysis.

### 3.5. Gene Differential Expression Analysis

**Figure 3:**
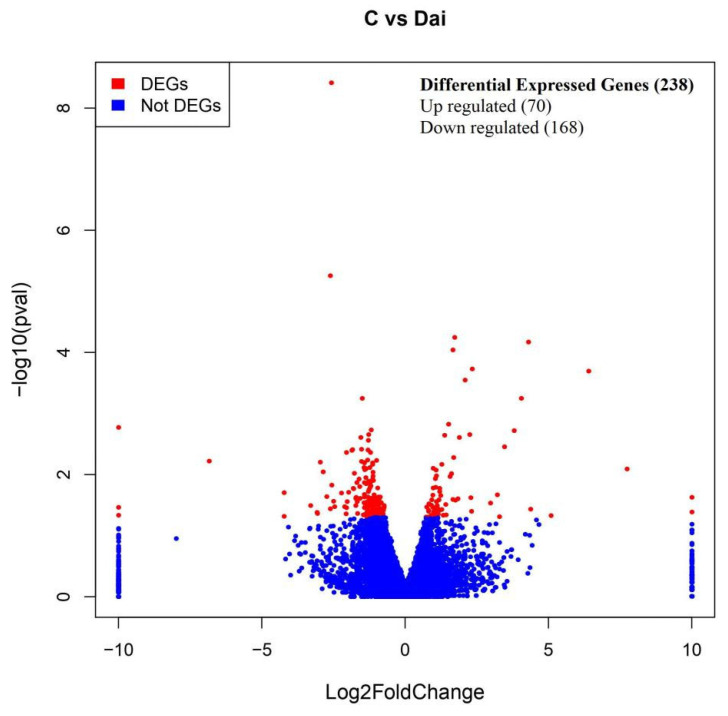
Volcano plot of the differentially expressed genes between the control and daidzein groups in the longissimus dorsi muscle. The red dots represent DEGs (*p* < 0.05), and the blue dots represent non-DEGs (*p* > 0.05).

In the LM samples, we identified 238 differentially expressed genes between the control group and the daidzein group (Figure 3). Among these DEGs, 70 were upregulated and 168 were downregulated in the daidzein group compared to the control group. Of the 238 DEGs, 13 were found to be related to lipid metabolism (FOSL1, DGKH, Gadd45G, GAL, SEMA3, TOB, FABP8, TRIB2, Nech1, and GSTA3) and connective tissue structure (CSTB and ACTN) (Table 7).

**Figure 4:**
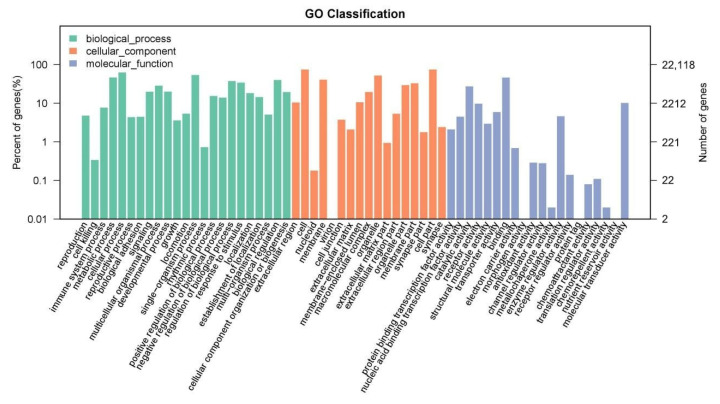
Gene ontology (GO) annotation of differentially expressed genes.

The DEGs were categorized into three gene ontology categories: biological process, molecular function, and cellular component (Figure 4). The top five molecular functional categories of DEGs between the control group and daidzein group included “binding”, “catalytic activity”, “molecular transducer activity”, “receptor activity”, and “enzyme regulator activity”. The top five biological processes enriched for DEGs included “cellular process”, “single-organism process”, “metabolic process”, “biological regulation”, and “regulation of biological process”. The top five cellular components enriched for DEGs included “cell”, “cell part”, “organelle”, “membrane”, and “membrane part”.

**Figure 5:**
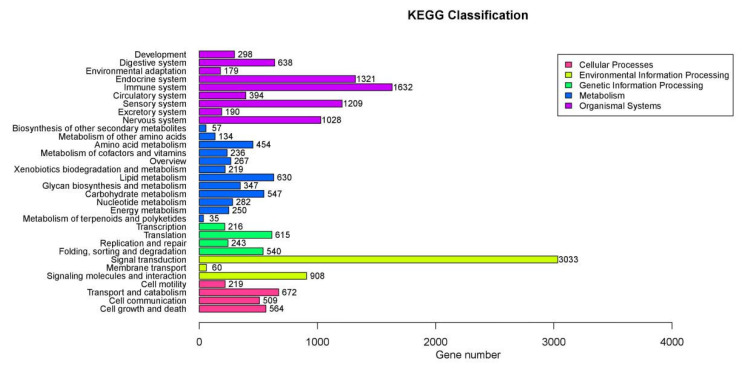
The bar graph of annotation classification in the KEGG pathway.

The Kyoto Encyclopedia of Genes and Genomes (KEGG) pathway enrichment analysis showed that the DEGs were significantly enriched in 23 signaling pathways (*p* < 0.05) (Table 8, Figure 5). In the 23 significantly enriched signaling pathways, the Notch signaling pathway (*p* = 0.023) and FoxO signaling pathway (*p* = 0.029 were closely related to lipid metabolism in animals, and regulation of the actin cytoskeleton was closely related to beef tenderness (*p* = 0.045).

The ordinate is the name of the KEGG pathway; the abscissa is the number of genes annotated in the signal pathway; the genes are divided into five branches according to the KEGG pathway: Cellular Processes, Environmental Information Processing, Genetic Information Processing, Metabolism, and Organismal Systems.

### 3.6. Confirmation of RNA-Seq Experiment

**Figure 6:**
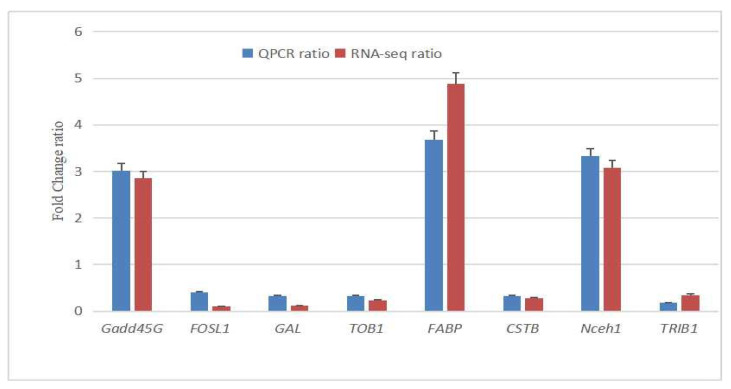
Comparing analysis of relative gene expression in the control and daidzein groups. Gadd45G, growth arrest and DNA damage gene 45; FOSL1, fos antigen-like 1; GAL, galanin and GMAP prepropeptide; TOB1, transducer of erbB−2 1; FABP, fatty acid binding protein 7; CSTB, cystatin B; Nceh1, neutral cholesterol ester hydrolase 1; TRIB1, tribbles pseudokinase 1.

Eight protein-coding DEGs (Gadd45G, FOSL1, GAL, TOB1, FABP, CSTB, Nceh1, and TRIB1) were randomly selected for validation. The results demonstrated a high degree of consistency between RNA-Seq and the qPCR data (Figure 6), confirming the reliability of the relative gene expression measurements obtained through RNA-Seq in this study.

## 4. Discussion

In the present study, the average THI during the feeding trial was 81.82, indicating that the experimental Jinjiang cattle were exposed to heat stress, as defined by the criteria reported by Armstrong [22]. Beef cattle primarily regulate body temperature through two key thermoregulatory mechanisms: evaporative cooling (panting and sweating) and conductive heat exchange. These physiological responses help reduce metabolic heat production by decreasing feed intake. Therefore, ADMI serves as a reliable indicator for assessing the health and performance of beef cattle under heat stress. Numerous studies have demonstrated that heat stress reduced dry matter intake in beef cattle, with feed intake showing a significantly negative correlation with environmental temperature [23,24]. In this study, daidzein supplementation significantly increased ADG in heat-stressed Jinjiang cattle, which was consistent with findings from previous research.

Leptin, a protein hormone predominantly secreted by adipose tissue, serves as a key regulator of energy homeostasis, neuroendocrine activity, and metabolic processes [25]. Research has indicated that heat stress elevates leptin concentrations in beef cattle [26]. The present study demonstrated that daidzein supplementation significantly decreased serum leptin levels compared to the control group. TC, a lipid metabolite in serum, is considered a fundamental metabolic parameter [27]. TC levels can reflect alterations in metabolic function and the body’s adaptation to external environmental conditions [28]. Studies have shown that heat stress significantly elevates serum TC levels in animals [29]. The current results show that daidzein significantly decreased TC concentrations in heat-stressed Jinjiang cattle. These results indicate that daidzein has the potential to ameliorate heat stress-induced endocrine dysfunction in beef cattle.

When beef cattle are exposed to heat stress, meat quality is significantly compromised after slaughter, often resulting in the occurrence of DFD meat [30]. In the current results, daidzein supplementation significantly decreased the L* value of LM in heat-stressed Jinjiang cattle, which suggests that daidzein can alleviate the effects of heat stress on beef meat color. Furthermore, the present findings indicate that adding daidzein to the diet significantly improved the marbling score and beef tenderness. However, daidzein did not significantly affect the IMF content of the longissimus dorsi muscle in heat-stressed Jinjiang cattle. Therefore, we hypothesized that daidzein might regulate the expression of genes related to differentiation of preadipocytes, thereby promoting a more uniform distribution of IMF in the longissimus dorsi muscle. This potential redistribution of fat may interfere with cross-linked collagen structures, ultimately enhancing beef tenderness. To explore this hypothesis, RNA-Seq was performed to elucidate the underlying mechanisms of daidzein on beef tenderness of the longissimus dorsi muscle in heat-stressed Jinjiang cattle. The results revealed that 238 genes were differentially expressed between the control and daidzein-treated groups (*p* < 0.05). These DEGs were found to be significantly involved in both preadipocyte differentiation and connective tissue restructuring.

The FoxO signaling pathway plays an important role in regulating the adipogenic differentiation of preadipocytes. Nakae et al. [31] reported that the FoxO gene acts as a negative regulatory factor, showing strong expression during the middle and late stages of preadipocyte differentiation, thereby inhibiting this process. Bastie [32] found that the FoxO protein could inhibit the metabolism of glycolysis and fat synthesis, reducing triglyceride synthesis. Sakamoto et al. [33] reported that the expression of the Gadd45 gene could be directly regulated by the FoxO signaling pathway and that the Gadd45 gene could promote preadipocyte differentiation by participating in cellular DNA methylation. In the present study, daidzein significantly downregulated the expression of PLK and TRAIL genes in the FoxO signaling pathway, thereby significantly inhibiting the FoxO signaling pathway’s activity, while significantly upregulating the expression of the Gadd45 gene. This suggests that daidzein promotes preadipocyte differentiation in the longissimus dorsi muscle of heat-stressed Jinjiang cattle through modulation of the FoxO signaling pathway.

The Notch signaling pathway plays a crucial role in regulating adipogenic differentiation [34]. Inhibition of the Notch signaling pathway could promote the adipogenic differentiation of preadipocytes [35]. The present results indicated that daidzein significantly downregulated the Notch gene expression, which was the key gene in the Notch signaling pathway, and significantly inhibited the Notch signaling pathway, thereby promoting the differentiation of preadipocytes in the longissimus dorsi muscle of heat-stressed Jinjiang cattle.

Daidzein significantly downregulated the expression of the *SEMA3* and *TOB* genes in the present study. The *SEMA3* and *TOB* genes were mainly responsible for the differentiation of MSCs [36,37]. The downregulated expression of the *SEMA3* and *TOB* genes could inhibit the differentiation of MSCs towards fibroblasts, while promoting their differentiation into preadipocytes. This, in turn, may enhance the differentiation of preadipocytes in the longissimus dorsi muscle of heat-stressed Jinjiang cattle.

This experiment also indicated that daidzein significantly regulated the expression of several genes related to fat synthesis in the longissimus dorsi muscle of heat-stressed Jinjiang cattle. Specifically, daidzein significantly upregulated the expression of the *FABP8* and *PON3* genes, while significantly downregulating the expression of the *TRIB1*, *DGKH,* and *Nceh1* genes. These regulatory effects suggested that daidzein could promote triglyceride synthesis in the longissimus dorsi muscle. Considering that daidzein significantly improved the marbling score, but had no significant effect on the IMF content in the longissimus dorsi muscle of heat-stressed Jinjiang cattle, we hypothesized that the experimental fattening period of 90 days might have been insufficient to induce a measurable change in IMF content. Following adipogenic differentiation of preadipocytes, triglyceride synthesis and subsequent deposition within adipocytes lead to an increase in adipocyte size, which is a critical process in the development of intramuscular fat in the longissimus dorsi muscle [38]. Therefore, the duration of this experiment may not have been long enough to observe a significant difference in IMF content.

Muscle fiber type, IMF content, and connective tissue structure were the main factors that affected beef tenderness [8]. Based on the findings of the current study and previous research [16], daidzein had no significant effect on the IMF content or muscle fiber types, but it significantly improved the marbling score and beef tenderness. Therefore, we hypothesized that daidzein might improve beef tenderness by changing the structure of connective tissue in the longissimus dorsi muscle of heat-stressed Jinjiang cattle.

To date, the molecular mechanism of fat deposits affecting beef tenderness is still unclear. Nishimura [39] speculated that fat deposits could disrupt the structure of connective tissue in muscle and reduce the mechanical strength of connective tissue, thus improving the tenderness of meat. This hypothesis has gradually attracted more and more attention from scholars, but unfortunately, no in-depth research reports are currently available. The present study showed that daidzein significantly downregulated the expression of the *CSTB* gene, which promoted the hydrolysis of cysteine by cysteine protease [40]. There are two main forms of covalent cross-linking of collagen molecules: the pyridine cross-linking between lysine and hydroxylysine [41], and the disulfide bonds based on cysteine [42]. Thus, the hydrolysis of cysteine could destroy the cross-linking of collagen and improve beef tenderness.

As a myofiber cross-linking protein [43], ACTN plays a role in maintaining muscle structure. The low expression of the *ACTN* gene in muscle fibers would contribute to improved beef tenderness. The results of this experiment showed that daidzein significantly downregulated the expression of the *ACTN* gene in the longissimus dorsi muscle of heat-stressed Jinjiang cattle. This downregulation may partially explain the observed improvement in beef tenderness following daidzein treatment.

## 5. Conclusions

In conclusion, daidzein supplementation in the diet significantly enhanced production performance and improved the meat quality of the longissimus dorsi muscle in heat-stressed Jinjiang cattle. These findings suggest that daidzein plays a positive role in relieving heat stress and improving beef quality in heat-stressed Jinjiang cattle. Nevertheless, it is important to acknowledge that the study focused solely on one breed of beef cattle. Therefore, further research is necessary to evaluate the effects of daidzein on production performance and meat quality across different breeds under heat stress conditions. The outcomes of this study provide a novel perspective for assessing the potential application of daidzein in ruminant production.

## Figures and Tables

**Table 1:** Components and nutritional composition of the basal diet (daidzein was included according to treatment).

Ingredients	%	Nutrient Level	%, MJ/kg
Corn	38.88	NE_mf_^3^/(MJ/kg)	6.85
Soybean meal	6.80	Crude protein	13.50
Cottonseed meal	4.80	Neutral detergent fiber	37.89
Rapeseed meal	3.20	Acid detergent fiber	20.95
Sunflower meal	1.52	Ca	1.02
DDGS ^1^	3.60	P	0.62
Limestone	0.20		
NaHCO_3_	0.70		
NaCl	0.30		
Premix ^2^	1.00		
Wheat straw	40.00		
Total	100.00		

^1^ DDGS, distillers’ dried grain with solubles. ^2^ The pre-mix provided the following nutrients per kg of diet: VA 30 000 000IU, VD3 1 000 000IU, VE 80 000IU, VK3 10 g, VB1 10 g, VB2 25 g, Cu 0.04 g, Fe 0.17 g, Zn 0.088 g, Mn 0.064 g, Co 0.02 g, and I 0.03 g. This pre-mix was purchased from Beijing Dabeinong Biotechnology Co., Ltd. (Beijing, China). ^3^ NEmf was calculated according to the Chinese Beef Cattle Feeding Standard (NY/T815–2004), while the other nutrient levels were measured values.

**Table 2:** Oligonucleotide primers used for quantitative real-time PCR.

Gene	PrimerBank ID	Orientation	Primer Sequences (5′−3′)	Product Size (bp)
GAPDH	126012538c3	Forward	GGTGATGCTGGTGCTGAG	168
		Reverse	GGTGTTGTTATACTTCTCGTGGTT	
Gadd45G	254553385c3	Forward	CTGCTGTGAGAACGACATTGA	183
		Reverse	CTCTCCTCGCAGAACAAACTG	
FOSL1	6753896a1	Forward	ATGTACCGAGACTACGGGGAA	140
		Reverse	CTGCTGCTGTCGATGCTTG	
GAL	118129967c1	Forward	AGAGGCAGCGTTATCCTGCTA	161
		Reverse	TCGCTAAATGATCTGTGGTTGTC	
TOB1	61676218c2	Forward	ATATGAAGGGCACTGGTATCCT	100
		Reverse	GGATGCCTGCTCGATCACG	
FABP	36054052c2	Forward	GCACATTCAAGAACACGGAGA	203
		Reverse	CACATCACCAAAAGTAAGGGTCA	
CSTB	6681071a1	Forward	AGGTGAAGTCCCAGCTTGAAT	196
		Reverse	GTCTGATAGGAAGACAGGGTCA	
Nceh1	142363800c1	Forward	ATGAGGTCGTCATGCGTCCTA	193
		Reverse	TGAAATTCAGCGCGATCAGAT	
TRIB1	146149168c2	Forward	GCTCGGCTCTTCAAGCAGATT	129
		Reverse	GCTTTCCAGTCTAAGCTGGGT	

GAPDH, glyceraldehyde−3-phosphate dehydrogenase; Gadd45G, growth arrest and DNA damage gene 45; FOSL1, fos antigen-like 1; GAL, galanin and GMAP prepropeptide; TOB1, transducer of erbB−2 1; FABP, fatty acid binding protein 7; CSTB, cystatin B; Nceh1, neutral cholesterol ester hydrolase 1; TRIB1, tribbles pseudokinase 1.

**Table 3:** Effects of daidzein on the growth performance in heat-stressed Jinjiang cattle.

Item ^1^	Treatment		SEM ^2^	*p*-Value
	**Control**	**Daidzein**		
IBW, kg	438	441	10.50	0.869
FBW, kg	514	522	9.691	0.736
ADG, kg/day	0.76	0.81	0.031	0.253
ADFI, kg/day	7.03	7.65	0.312	0.022
FCR	9.25	9.44	1.403	0.542

^1^ BW, body weight; ADG, average daily gain; AMDI, average dry matter intake; FCR, feed conversion rate. ^2^ SEM, standard error of the mean.

**Table 4:** Effects of daidzein on the serum biochemical parameters in heat-stressed Jinjiang cattle.

Item ^1^	Treatment		SEM ^2^	*p*-Value
	Control	Daidzein		
TC (mmol/L)	3.37	2.68	0.118	0.031
TG (mmol/L)	0.46	0.37	0.061	0.562
FFA (µmol/L)	506.35	591.34	25.23	0.011
Glucose (mmol/L)	11.82	9.45	1.920	0.610
TP (g/L)	73.42	80.31	4.112	0.695
Albumin (g/L)	29.32	28.16	1.221	0.894
HDL-C (mmol/L)	2.06	1.99	0.204	0.415
LDL-C (mmol/L)	0.85	0.89	0.052	0.509
Urea (mmol/L)	4.89	5.83	0.298	0.384
ALT (U/L)	24.70	38.05	1.033	0.019
AST (U/L)	76.13	82.11	2.188	0.778
T-SOD (U/mL)	155.23	149.58	5.328	0.225
MDA (nmol/mL)	5.66	5.33	0.522	0.298
GSH-PX (µmol/L)	877.43	879.12	19.661	0.853
T-AOC (U/mL)	13.11	14.23	1.002	0.198
IgA (g/L)	0.92	1.01	0.011	0.685
IgM (g/L)	0.79	0.82	0.034	0.711
IgG (g/L)	8.87	8.69	0.118	0.220
T3 (ng/mL)	2.93	2.89	0.118	0.125
T4 (ng/mL)	76.01	77.22	3.431	0.226
COR(ng/mL)	50.66	48.87	2.172	0.086
GH (ng/mL)	4.88	4.99	0.362	0.552
Insulin (ng/mL)	17.11	18.79	3.057	0.223
Leptin (ng/mL)	6.61	5.49	0.238	0.042
Adiponectin (ng/mL)	1.53	1.33	0.557	0.239

^1^ TC, total cholesterol; TG, triglyceride; FFA, free fatty acid; TP, total protein; HDL-C, high-density lipoprotein cholesterol; LDL-C, low-density lipoprotein cholesterol; ALT, glutamic-pyruvic transaminase; AST, glutamic-oxalacetic transaminase; T-SOD, total superoxide dismutase; MDA, malondiadehyde; GSH-PX, glutathione peroxidase; T-AOC, total antioxidant capacity; T3, triiodothyronine; T4, tetraiodothyronine; COR, cortisol; GH, growth hormone. ^2^ SEM = standard error of the mean (*n* = 10 cattle per treatment).

**Table 5:** Effects of daidzein on the meat quality in the LM of heat-stressed Jinjiang cattle.

Item ^1^	Treatment		SEM ^2^	*p*-Value
	Control	Daidzein		
Marbling score	2.51	3.58	0.221	0.032
pH	5.23	5.34	0.019	0.368
Shear force (kg·f)	3.62	3.04	0.215	0.042
Drip loss (g/kg)	18.9	18.1	0.688	0.239
Cooking loss (g/kg)	309.21	322.53	8.99	0.321
L*	47.33	43.59	0.531	0.032
a*	22.8	22.3	0.441	0.821
b*	13.1	12.8	0.222	0.924
Chemical composition				
Moisture (g/kg)	690.12	692.29	3.88	0.774
Ash (g/kg)	18.82	18.94	0.208	0.653
Crude protein (g/kg)	212.32	218.01	4.99	0.767
Intramuscular fat (g/kg)	82.41	83.55	4.12	0.847

^1^ L*, lightness; a*, redness; b*, yellowness. ^2^ SEM = standard error of the mean (*n* = 4 cattle per treatment).

**Table 6:** Summary statistics for sequence quality and alignment information of eight longissimus dorsi muscle samples in the two groups.

Sample Name	Control1	Control2	Control3	Control4	Daizein1	Daizein2	Daizein3	Daizein4
Raw reads	52,012,160	50,412,734	51,046,628	51,157,174	59,376,844	54,566,356	52,055,804	55,333,001
Clean reads	51,856,040	50,213,021	50,881,106	50,983,389	59,197,642	54,376,305	51,883,297	55,152,415
Valid ratio%	99.70	99.60	99.68	99.01	99.70	99.65	99.67	99.67
Q30(%)	95.71	95.27	95.65	95.54	95.70	95.44	95.63	95.59
GC(%)	50.34	52.69	51.43	51.49	52.78	52.24	50.56	51.86
Total reads	51,702,096	50,016,312	50,717,666	50,812,025	59,021,122	54,188,550	51,713,124	54,974,265
Total reads mapped	41,029,142	41,948,839	40,913,443	41,297,141	48,993,403	46,984,163	40,304,165	45,427,244
Multiple mapped	1,776,858	2,136,467	2,164,276	2,025,867	2,810,836	2,134,347	1,763,899	2,236,361
Uniquely mapped	39,252,284	39,812,372	3,8749,167	39,271,274	46,182,567	44,849,816	38,540,266	43,190,883
Mapping rate (%)	79.36	83.87	80.67	81.30	83.01	86.70	77.94	82.55

**Table 7:** Differentially expressed genes related to beef tenderness and lipid metabolism.

Gene ID	Gene Name	*p*-Value	Expression Trend
ENSBTAG00000006194	*FOSL1*	0.032	Down
ENSBTAG00000013879	*DGKH*	0.039	Down
ENSBTAG00000003033	*Gadd45G*	0.002	Up
ENSBTAG00000009393	*GAL*	0.042	Down
ENSBTAG00000018307	*SEMA3*	0.025	Down
ENSBTAG00000002749	*TOB1*	0.033	Down
ENSBTAG00000000071	*FABP8*	0.024	Up
ENSBTAG00000023179	*TRIB1*	0.006	Down
ENSBTAG00000020073	*Nceh1*	0.009	Up
ENSBTAG00000021516	*GSTA3*	0.001	Up
ENSBTAG00000011215	*ACTN*	0.049	Down
ENSBTAG00000020764	*CNN2*	0.023	Down
ENSBTAG00000014718	*CSTB*	0.004	Down

**Table 8:** Classification of DEGs according to the KEGG pathway enrichment analysis.

Pathway Name	Input Number	Background Number	*p*-Value
HTLV-I infection	14	271	0.001
Graft-versus-host disease	6	51	0.001
Herpes simplex infection	11	199	0.001
Type I diabetes mellitus	6	58	0.001
Natural killer cell mediated cytotoxicity	9	146	0.001
Allograft rejection	6	61	0.001
Endocytosis	10	193	0.001
Autoimmune thyroid disease	6	74	0.001
Viral myocarditis	6	80	0.001
Antigen processing and presentation	6	84	0.001
Cell adhesion molecules (CAMs)	8	155	0.001
Epstein–Barr virus infection	9	198	0.001
Apoptosis	5	78	0.004
Glycosaminoglycan biosynthesis	2	14	0.015
Phosphatidylinositol signaling system	4	77	0.021
Viral carcinogenesis	8	250	0.021
Notch signaling pathway	3	45	0.023
FoxO signaling pathway	5	127	0.029
Phagosome	6	172	0.030
Glutathione metabolism	3	50	0.031
Bisphenol degradation	1	3	0.041
Regulation of actin cytoskeleton	5	205	0.045

## Data Availability

The data presented in this study are available on request from the corresponding author. The data are not publicly available due to institutional privacy policies.
